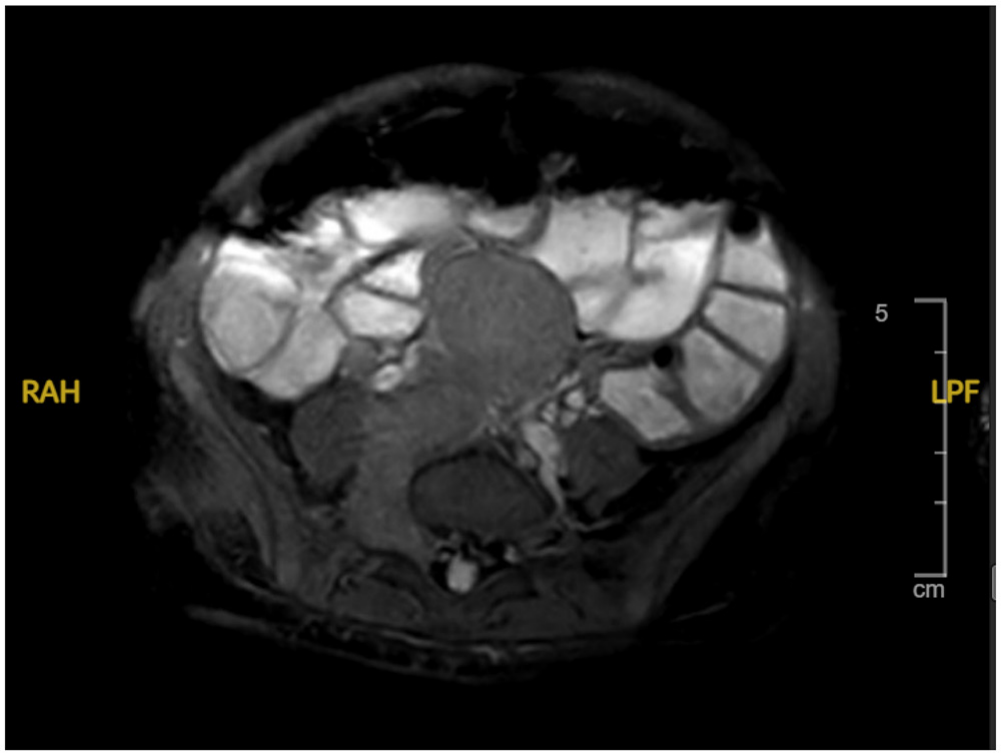# Poster Session I - A82 VIP-SECRETING NEUROBLASTOMA AS A RARE CAUSE OF PERSISTENT SECRETORY DIARRHEA IN INFANCY

**DOI:** 10.1093/jcag/gwaf042.082

**Published:** 2026-02-13

**Authors:** A Aljaafari, P Church

**Affiliations:** University of Toronto, Toronto, ON, Canada; University of Toronto, Toronto, ON, Canada

## Abstract

**Background:**

Secretory diarrhea in infancy is rare and can be associated with paraneoplastic syndromes such as vasoactive intestinal peptide (VIP)–secreting neuroblastoma. **Less than 1% of patients with neuroblastoma have clinical evidence of VIP secretion, and in these rare cases, chronic secretory diarrhea may be one of the earliest presenting symptoms.** Delay in recognition may result in severe electrolyte derangements and life-threatening complications.

**Aims:**

To report a case of an infant presenting with intractable watery diarrhea ultimately attributed to a VIP-secreting neuroblastoma.

**Methods:**

A 12-month-old girl presented with persistent watery, non-bloody diarrhea occurring several times per day, accompanied by poor oral intake, lethargy, vomiting, and ∼12% weight loss over 10 days. Initial admission identified adenovirus on stool PCR with partial improvement after correction of hypokalemia. Symptoms recurred shortly after discharge, with worsening diarrhea, severe hypokalemia (2.2 mmol/L), metabolic acidosis with a normal anion gap (4 mmol/L), and prolonged QTc, requiring pediatric intensive care for electrolyte repletion and monitoring.

**Results:**

Despite stabilization, she continued to pass 4–6 watery stools daily with persistent electrolyte losses and dependence on intravenous potassium supplementation. Stool electrolytes demonstrated a secretory pattern. Endoscopy was normal macroscopically and microscopically. MRI revealed a multilobulated retroperitoneal mass centered at the organ of Zuckerkandl with vascular encasement and foraminal extension, consistent with neuroblastoma. Urinary vanillylmandelic acid (16.4) and homovanillic acid (43.9) were elevated. Metaiodobenzylguanidine (MIBG) scintigraphy confirmed an avid abdominal lesion without distant metastases.

Despite trials of **loperamide, atropine, and continuous octreotide infusion**, stool output remained unchanged. In view of her ongoing weight loss and sustained dependence on intravenous fluid supplementation, **total parenteral nutrition (TPN)** was initiated to provide adequate nutritional support. She was subsequently transferred to the Oncology service, where **systemic chemotherapy was commenced and is ongoing at the time of this report.**

**Conclusions:**

This case highlights a rare presentation of VIP-secreting neuroblastoma in infancy, characterized by refractory secretory diarrhea and profound electrolyte disturbances, including normal anion gap metabolic acidosis. Recognition of this association through appropriate imaging and biochemical testing is essential for timely diagnosis and initiation of disease-directed therapy in children with unexplained persistent diarrhea.

**Funding Agencies:**

None